# Supplementary material for: Microbiota, Microbial Metabolites, and Barrier Function in A Patient with Anorexia Nervosa after Fecal Microbiota Transplantation
Source: Microorganisms. 2019 Sep 10;7(9):338. doi: 10.3390/microorganisms7090338 (PMC6780752; doi:10.3390/microorganisms7090338)
Supplement: Supplementary file 1 [file microorganisms-07-00338-s001.pdf]

microorganisms

Supplementary Materials and Methods

NMR- and MS-Based Metabolomic Analysis of the Stool Samples

Sample Preparation

Stool samples for NMR and MS analyses were obtained by extraction of approx. 100 mg aliquot with 1 mL of water (MS-LC grade). The mixture was then vortexed till completely homogenized and centrifuged at 6.000 rpm. The supernatant was filtered through a syringe filter (Whatman 25 mm GD/X PP, pore size 0.2 μm). The filter was then rinsed with 0.5 mL H_2O. In the case of NMR samples, 1 mL of mixture methanol:dichloromethane (2:1) (MS-LC grade, biotech. grade) was added to the collected filtrate. The mixture was vortexed and centrifuged at 14.000 rpm for 30 min (5 °C). The upper hydrophilic phase was transferred to a fresh tube and evaporated on Speedvac (35 °C) and stored at – 80 °C till analysis. The dried matter was re-dissolved in 450 μL D_2O and 50 μL phosphate buffer (1.5 M KH_2PO_4 in D_2O containing 2 mM NaN_3 and 0.1% (w/v) trimethylsilyl propionic acid (TSP), pH 7.4) and transferred to a 5-mm NMR tube.

In the case of MS samples, 200 μL aliquot of water extract was transported to a fresh tube and underwent derivatization following user´s manual instruction in the EZ:fast kit (Phenomenex, USA). In the workflow, it was necessary to optimize the pH of the extract by the addition of 1 μL formic acid to reach pH suitable for solid phase extraction.

NMR Analysis

The NMR spectra were recorded on a 600 MHz Bruker Avance III spectrometer (Bruker BioSpin, Rheinstetten, Germany) equipped with a 5-mm TCI cryogenic probe head. All NMR experiments were performed at 300 K. Standard ^{1}H NMR spectra were acquired using nuclear Overhauser effect spectroscopy (NOESY) pulse sequence with following acquisition parameters: number of scans (NS) = 256, 64k of data points (TD), spectral width (SW) of 20 ppm, relaxation delay (D1) of 4 s. The resonance of water was suppressed by presaturation during the relaxation delay. J-resolved experiment with presaturation (NS=4, SW=16 ppm, TD=8k, number of increments=40, SW=78.125 Hz in the indirect dimension, and relaxation delay=2 s) was performed to facilitate the identification of metabolites.

The raw spectral data were processed using TopSpin 3.6 software (Bruker BioSpin, Rheinstetten, Germany). The free induction decays were multiplied by an exponential window function (LB = 0.3 Hz). The spectra were automatically phased, baseline corrected and referenced to TSP (0.0 ppm). Next, the spectral baselines were adjusted, the spectra were binned and normalized, all procedures using MATLAB software (‘MATLAB version 9.2 (R2017a).

The calculated intensities were normalized by probabilistic quotient normalization [1] with a group of samples from the patient as a reference. The metabolites´ identification was carried out using the Chenomx NMR Suite 7.7 database (Chenomx Inc., Edmonton, AB, Kanada). Signals of short-chain fatty acids acetate (methyl group at 1.92 ppm), butyrate (methyl group at 0.90 ppm), and propionate (methyl group at 1.06 ppm) were quantified using normalized intensities of corresponding signals.

MS Analysis

The analysis was conducted in SRM mode using TSQ Quantum Access Max mass spectrometry (Thermo Fisher Scientific, Inc., USA). The MS/MS parameters were optimized using direct infusion (10 mg/L in the mobile phase, 20 μL/min) in positive ionization mode of serotonin, 5-hydroxytryptophan, tryptophan and kynurenine standards (AlfaAesar, UK). The parameters are shown in Table S1. The calibration curve was constructed in the concentration range from 10 to 1000 pmol/mL. The capacity of the solid phase extraction column was tested by extraction of 50, 100 and 200 mg of feces.

Table S1. SRM parameters.

Metabolites	parent ion	product ions	collision energy (V)	tube lens voltage (V)	retention time (min)
Tryptophan	333.0	245, 273	17, 12	68	9.1
5-hydroxytryptophan	435.1	201, 375	29, 12	69	11.6
Kynurenine	423.0	146, 260	33, 15	64	15.5
Serotonin	349.1	160, 203	30, 17	60	8.4

TSQ MS detector was equipped by HESI-II probe run under the following conditions: spray voltage +2250 V, vaporizer temperature 320 °C, sheath gas pressure 34 arbitrary units (AU), auxiliary gas pressure 15 AU, ion sweep gas pressure 11.2 AU, collision gas (Ar) pressure 1.0 mTorr, capillary temperature 320 °C. Skimmer offset voltage was not used. Data were processed by software ThernoXcalibur (Thermo Fisher Scientific, Inc., USA). The peaks area was normalized by the area of internal standard and raw stool sample weight.

Quantification of Intestinal Microbiota by qPCR

The amplifications were performed in 25 ul reaction mixtures (Sybr green master mix, Bio-Rad) containing the same amount of gDNA template in each sample (40 ng). The sequences and origins of primers are shown in Table S2 [2-4]. Cycling parameters were as follows: 4 min at 94 °C, 35 cycles of 10 s at 94 °C, 25 s at 60 °C, and 35 s at 72 °C, and a final extension for 7 min at 72 °C. Amplifications were carried out in duplicates. The specificities of all used primers and amplified products were checked by melting curve and by sequencing. The PCR fragments of corresponding genes were ligated into the pCR2.1-TOPO cloning vector (Life Technologies) with subsequent transformation into chemically competent *E. coli* cells (Neb 5-alpha *E. coli*, Bio Labs). To obtain their linear form, plasmids were cut with BamH1 restriction endonuclease (37 °C, 12 hours). Plasmids were then purified by PureLink Quick Plasmid Kit (Thermo Fisher Scientific Baltics, UAB. Vilnius, Lithuania). The experiments were performed three times from DNA isolated from 3 distinct parts of the stools.

High Throughput Sequencing of Microbiota Composition

Total extracted gDNA from stool samples was used for high throughput sequencing (Miseq platform, Illumina) of the bacterial V4 region of 16S rRNA gene and fungal ITS region. Two sets of specific primers with barcodes (Table S2) were used in PCR using KAPA 2G Robust Hot Start DNA Polymerase (Kapa Biosystems) were carried out with 25 and 27 cycles, respectively [5,6]. The PCR products were purified and normalized with the SequalPrep™ Normalization Plate Kit (ThermoFisher Scientific). Triplicates of the amplicons were pooled and ligated with sequencing adapters (TruSeq DNA PCR-free LT Sample Preparation Kit, Illumina), pooled in equimolar concentrations, and sequenced. The library was validated by a KAPA Library Quantification Kit (Illumina). The amplicons were sequenced on an Illumina MiSeq using a Miseq Reagent Kit v3 (Illumina).

The amplicon sequencing data were processed with SEED v2.1 [7] applying standard procedures such as through quality control and data filtering, clustering analysis, and diversity determination. Pair-end reads were joined using fastq-join [8]. Chimeric sequences were detected using algorithm

UCHIME, deleted, and clustered using UPARSE at a 97% similarity level, both of which were conducted with USEARCH 8.1.1861 [9]. The most abundant sequences were chosen as one representative strain per cluster, and the closest hits at the genus level were identified using the RDP database for bacteria [10] or UNITE database for fungi [11]. The dataset obtained in this study was deposited in the NCBI Sequence Read Archive (raw demultiplexed sequencing data with sample annotations, PRJNA542274). Singletons were excluded from all analyses.

Table S2. Primers used in qPCR and for HTS.

Name	Sequence (5′–3′)	Target	References
BAC	CGGCAACGAGCGCAACCC	univ. bact. 16S rRNA	Denman and McSweeney 2006
BAC	CCATTGTAGCACGTGTGTAGCC		
AM1129F	CAGCACGTGAAGGTGGGGAC	*A. muciniphila* 16S rRNA	Collado et al. 2007
AM1437R	CCTTGCGGTTGGCTTCAGAT		
MS534F	CCGGGTATCTAATCCGGTTC	*M. smithii* 16S rRNA	Dridi et al. 2009
MS656R	CTCCCAGGGTAGAGGTGAAA		
515F	xxxxxxxGTGCCAGCMGCCGCGGTAA	univ. bact. 16S rRNA	Caporoso et al. 2011
806R	xxxxxxxGGACTACHVGGGTWTCTAAT		
gITS7	xxxxxxxGTGARTCATCGARTCTTTG	univ. fungal ITS DNA	Ihrmark et al. 2012
gITS4	xxxxxxxTCCTCCGCTTATTGATATGC		

X represents a barcode base with linkers.

Supplementary Results

Taxonomic Composition of the Microbial Community

To determine the composition of microbiota in stool samples, isolated gDNA was used for the microbiome analysis by HTS. All sequences with mismatches in tags were removed from the dataset, and a total of 1,261,800 bacterial and 1,193,130 fungal sequences were retained after the removal of low-quality sequences (with mean Phred quality score threshold of 30) and sequences shorter than 200 bases. It yielded 1,088,645 bacterial and 906,470 fungal sequences which were clustered to operational taxonomic units (OTUs) with 97% similarity threshold (166,165 bacterial and 1,063 fungal chimeric sequences and 2,975 bacterial and 406 fungal singletons were excluded during this step). The 1,153 bacterial OTUs were classified into 8 phyla and 156 genera. The 754 fungal OTUs were classified into 5 phyla and 270 genera. Most of the OTUs were identified at the genus level; however, identification down to the species level was impossible owing to database limitations. The total number and proportional distribution of OTUs among different phyla are shown in Figure 3.

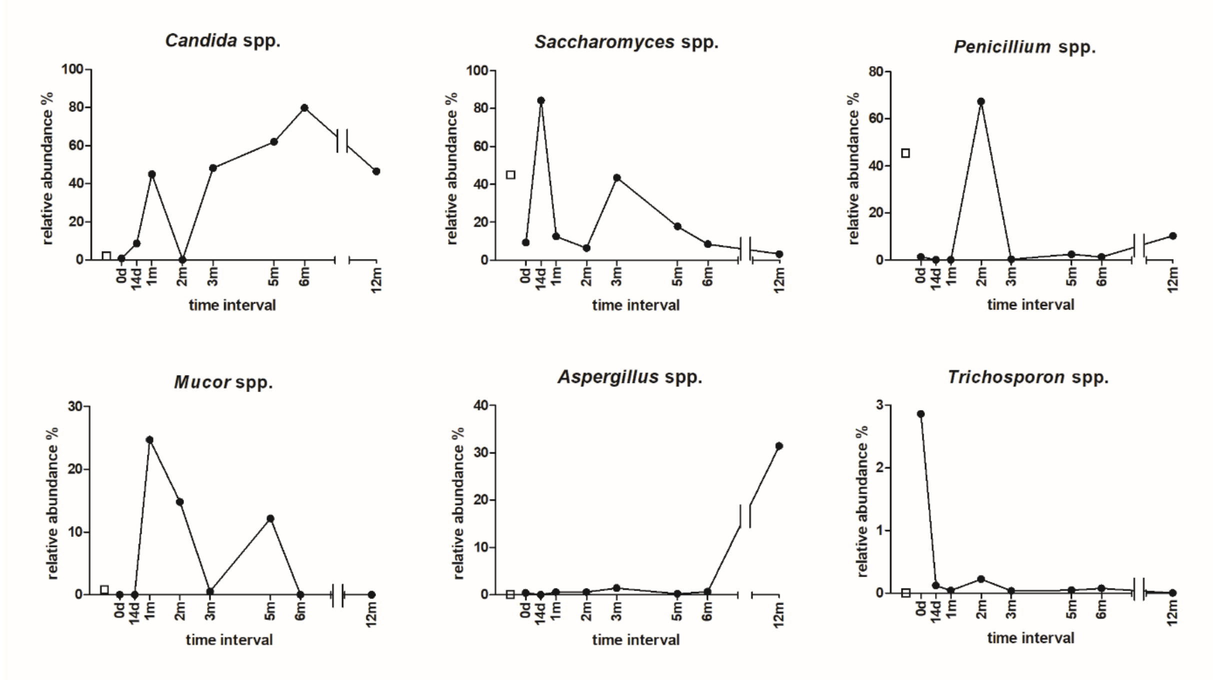

Figure 1. The relative abundance of assorted fungal species present in stool samples. Patient´s values are represented by black circles, donor value is represented by the empty square.

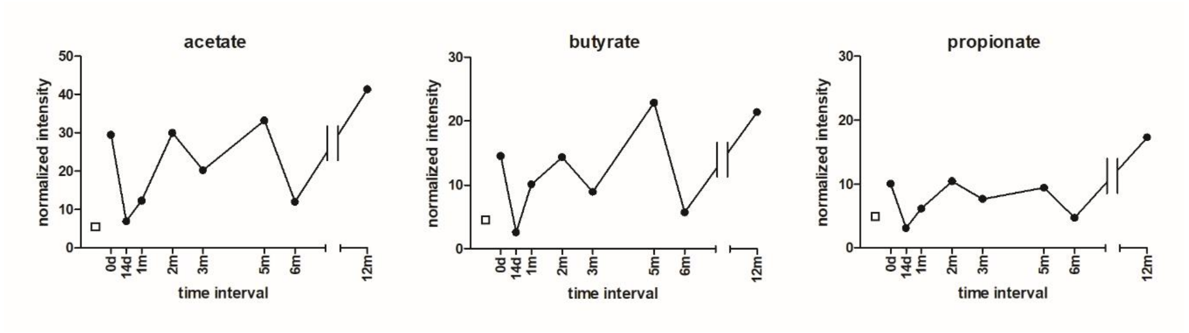

Figure 2. Acetate, butyrate, and propionate levels in stool samples.

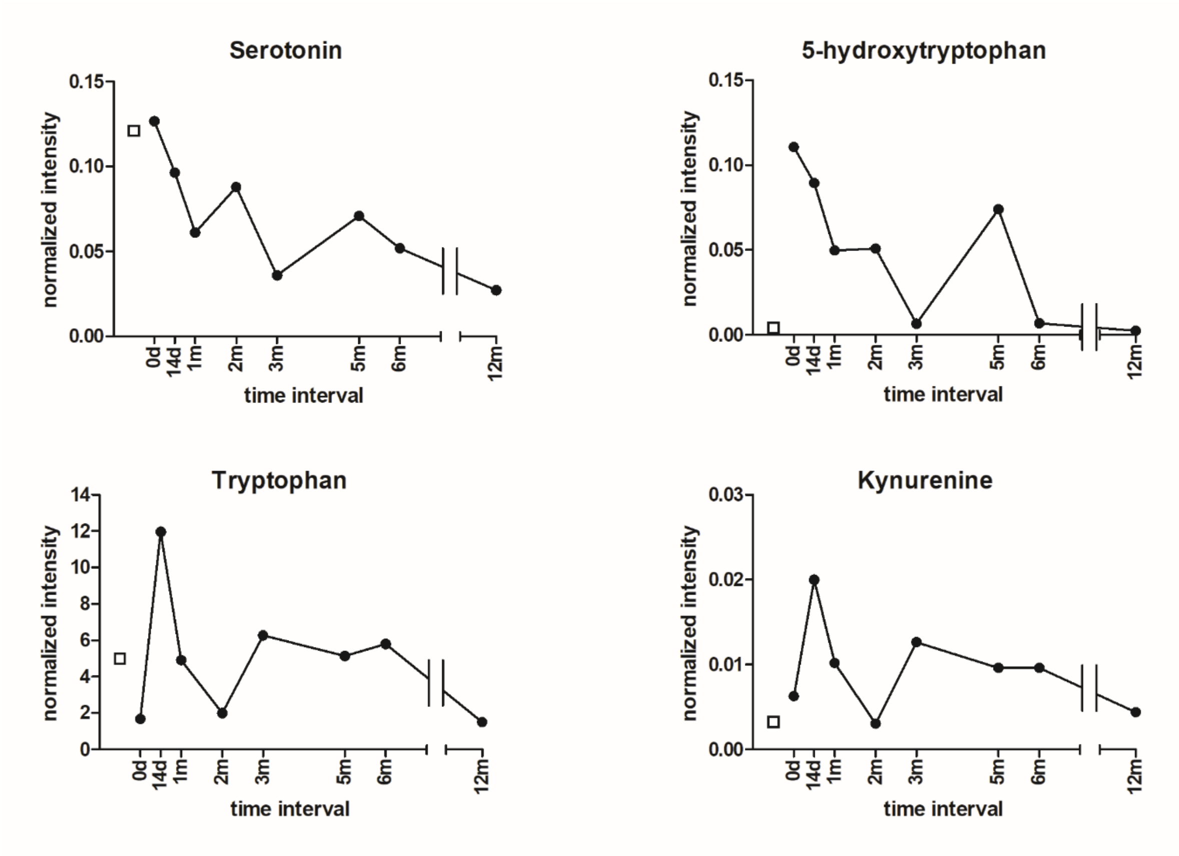

Figure 3. Serotonin, 5-hydroxytryptophan, kynurenine, and tryptophan levels in stool samples.

References

1. Dieterle, F.; Ross, A.; Schlotterbeck, G.; Senn, H. Probabilistic quotient normalization as robust method to account for dilution of complex biological mixtures. Application in 1H NMR metabonomics. *Anal Chem* **2006**, *78*, 4281-4290, doi:10.1021/ac051632c.
2. Collado, M.C.; Derrien, M.; Isolauri, E.; de Vos, W.M.; Salminen, S. Intestinal integrity and *Akkermansia muciniphila*, a mucin-degrading member of the intestinal microbiota present in infants, adults, and the elderly. *Appl Environ Microbiol* **2007**, *73*, 7767-7770, doi:10.1128/AEM.01477-07.
3. Denman, S.E.; McSweeney, C.S. Development of a real-time PCR assay for monitoring anaerobic fungal and cellulolytic bacterial populations within the rumen. *FEMS Microbiol Ecol* **2006**, *58*, 572-582, doi:10.1111/j.1574-6941.2006.00190.x.
4. Dridi, B.; Henry, M.; El Khechine, A.; Raoult, D.; Drancourt, M. High prevalence of *Methanobrevibacter smithii* and *Methanosphaera stadtmanae* detected in the human gut using an improved DNA detection protocol. *PLoS One* **2009**, *4*, e7063, doi:10.1371/journal.pone.0007063.
5. Caporaso, J.G.; Lauber, C.L.; Walters, W.A.; Berg-Lyons, D.; Lozupone, C.A.; Turnbaugh, P.J.; Fierer, N.; Knight, R. Global patterns of 16S rRNA diversity at a depth of millions of sequences per sample. *Proc Natl Acad Sci U S A* **2011**, *108 Suppl 1*, 4516-4522, doi:10.1073/pnas.1000080107.
6. Ihrmark, K.; Bodeker, I.T.; Cruz-Martinez, K.; Friberg, H.; Kubartova, A.; Schenck, J.; Strid, Y.; Stenlid, J.; Brandstrom-Durling, M.; Clemmensen, K.E., et al. New primers to amplify the fungal ITS2 region--evaluation by 454-sequencing of artificial and natural communities. *FEMS Microbiol Ecol* **2012**, *82*, 666-677, doi:10.1111/j.1574-6941.2012.01437.x.
7. Vetrovsky, T.; Baldrian, P.; Morais, D.; Berger, B. SEED 2: a user-friendly platform for amplicon high-throughput sequencing data analyses. *Bioinformatics* **2018**, 10.1093/bioinformatics/bty071, doi:10.1093/bioinformatics/bty071.
8. Aronesty, E. Comparison of sequencing utility programs. *Open Bioinformatics J* **2013**, *7*, 1-8.

9. Edgar, R.C. UPARSE: highly accurate OTU sequences from microbial amplicon reads. *Nat Methods* **2013**, *10*, 996, doi:10.1038/Nmeth.2604.
10. Cole, J.R.; Wang, Q.; Fish, J.A.; Chai, B.L.; McGarrell, D.M.; Sun, Y.N.; Brown, C.T.; Porras-Alfaro, A.; Kuske, C.R.; Tiedje, J.M. Ribosomal Database Project: data and tools for high throughput rRNA analysis. *Nucleic Acids Res* **2014**, *42*, D633-D642.
11. Nilsson, R.H.; Larsson, K.H.; Taylor, A.F.S.; Bengtsson-Palme, J.; Jeppesen, T.S.; Schigel, D.; Kennedy, P.; Picard, K.; Glockner, F.O.; Tedersoo, L., et al. The UNITE database for molecular identification of fungi: handling dark taxa and parallel taxonomic classifications. *Nucleic Acids Res* **2019**, *47*, D259-D264, doi:10.1093/nar/gky1022.
